# Supplementary material for: Circadian Misalignment Induced by Chronic Night Shift Work Promotes Endoplasmic Reticulum Stress Activation Impacting Directly on Human Metabolism
Source: Biology (Basel). 2021 Mar 5;10(3):197. doi: 10.3390/biology10030197 (PMC7998626; doi:10.3390/biology10030197)
Supplement: Supplementary file 1 [file biology-10-00197-s001.pdf]

Supplementary Files:

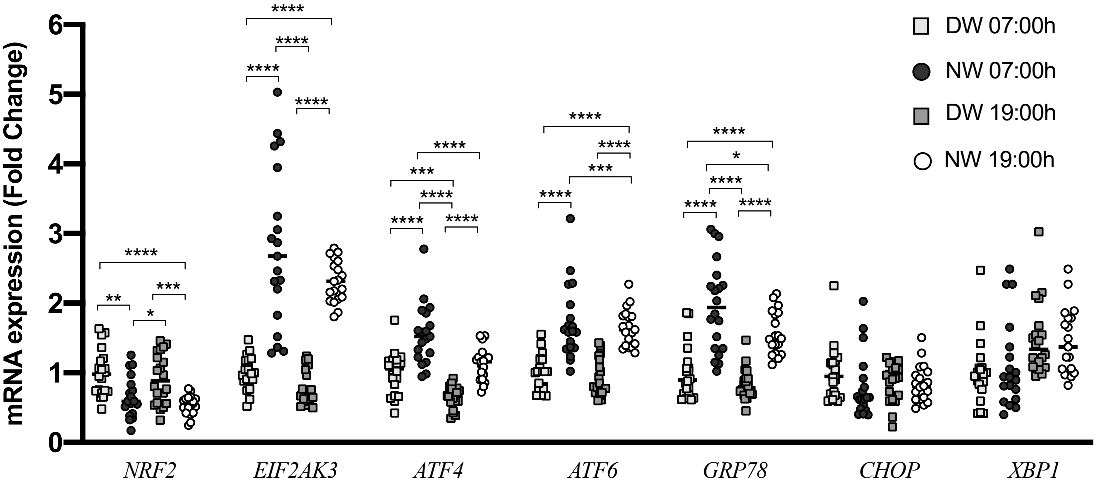

Supplementary Figure S1. Determination of the expression of genes related to endoplasmic reticulum stress in P.B.M.C. samples of day and night workers at 0700h and 1900h normalized by DW at 07:00h. Gene expression was evaluated by RT-qPCR. Data are presented as dot plot with mean. Unpaired, one-tailed t-test. *P<0.05; **P < 0.01; ***P = 0.001; ****P < 0.001.

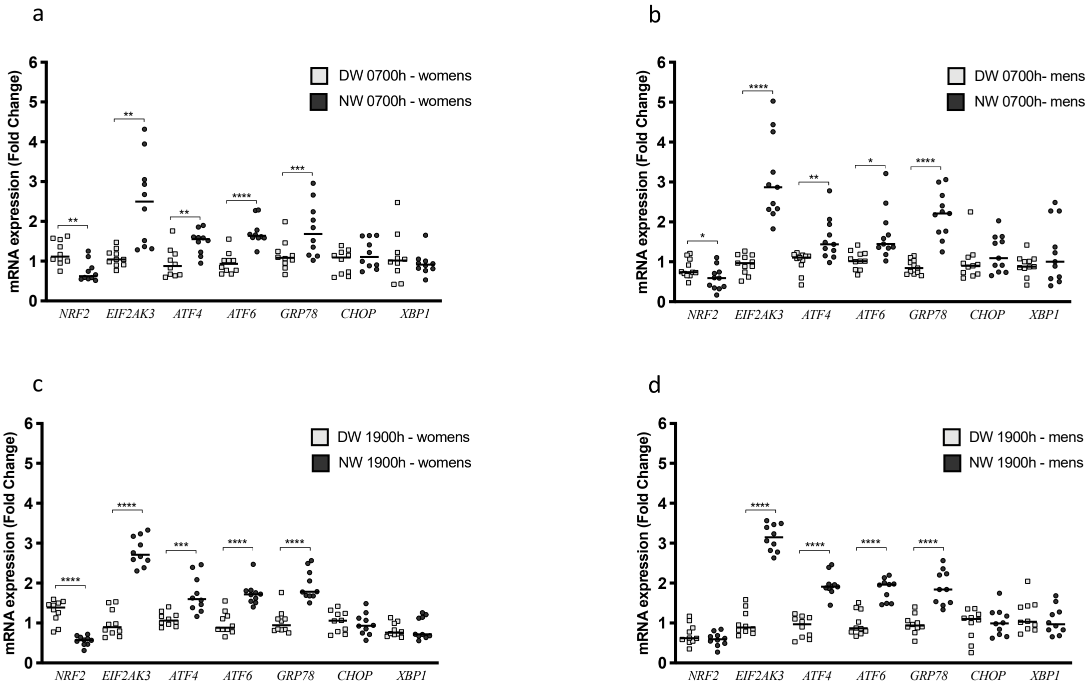

Supplementary Figure S2. Gene expression related of Endoplasmic Reticulum Stress genes in PBMC samples of day and night workers collected at 0700h and 1900h. A. Gene expression of women (n= 10) collected at 0700h. B. Gene expression of men (n=10) collected at 0700h. C. Gene expression of women (n= 10) collected at 1900h. D. Gene expression of men (n= 10) collected at 1900h. Gene expression was evaluated by RT-qPCR. Data are presented as dot plot with mean. Unpaired, one-tailed t-test. *P<0.05; **P < 0.01; ***P = 0.001; ****P < 0.001.

Supplementary Table S1. Sequences of the oligonucleotides. Oligonucleotides used for the analysis of expression in PBMC samples.

Gene	*Forward*	*Reverse*
CLOCK	TGCGAGGAACAATAGACCCAA	ATGGCCTATGTGTGCGTTGTA
BMAL	TGCAACGCAATGTCCAGGAA	GGTGGCACCTCTTAATGTTTTCA
CRY1	TTGGAAAGGAACGAGACGCAG	CGGTTGTCCACCATTGAGTT
PER1	GCCAACCAGGAATACTACCAGC	GTGTGTACTCAGACGTGATGTG
NRF2	TTCCCGGTCACATCGAGAG	TCCTGTTGCATACCGTCTAAATC
EIF2AK3	TGTCGCCAATGGGATAGTGACGAA	AATCCGGCTCTCGTTTCCATGTCT
ATF4	GGGTTCTCCAGCGACAAGGCTAAG	AACAGGGCATCCAAGTCGAACTC
ATF6	ATGTCTCCCCTTTCCTTATATGGT	AAGGCTTGGGCTGAATTGAA
GRP78	CATCACGCCGTCCTATGTCG	CGTCAAAGACCGTGTTCTCG
CHOP	GGAGAACCAGGAAACGGAAAC	TCTCCTTCATGCGCTGCTTT
XBP1	TGGCCGGGTCTGCTGAGTCCG	ATCCATGGGAAGATGTTCTGG
GAPDH	AGGGCTGCTTTTAACTCTGGT	CCCCACTTGATTTTGGAGGGA
18S	GGCCCTGTAATTGGAATGAGTC	CCAAGATCCAACTACGAGCTT

Supplementary Table S2 - Correlation between body weight and blood metabolites.

	**Weight vs. Glucose**	**Weight vs. Hb1ac**	**Weight vs. Cholesterol**	**Weight vs. Triglycerides**	**Weight vs. HDL**	**Weight vs. LDL**	**Weight vs. CRP**
**Day worker**	r=0,1106<br>p=0,642	r=0,1881<br>p=0,427	r=0,1055<br>p= 0.658	r=0,3357<br>p=0,147	r=-0,05999<br>p=0,801	r=0,1328<br>p=0,576	r=-0,0385<br>p=0,872
**Night worker**	r=0,2709<br>p=0,248	r=-0,2765<br>p=0,238	r=0,0836<br>p=0,725	**r=-0,5135**<br>**p=0,020**	r=0,2064<br>p=0,382	r=-0,0036<br>p=0,987	r=0,0764<br>p=0,748

Supplementary table S3. - Correlation between ERS genes and CLOCK genes at 0700h and 1900h.

	0700h			1900h	
	Day worker	*Night worker*		*Day worker*	*Night worker*
**NRF2 vs CLOCK**	r=0,1531<br>p=0,742	r=0,4256<br>p=0,345	**NRF2 vs CLOCK**	r=0,5012<br>p=0,128	r=0,4456<br>p=0,238
**EIF2AK3 vs CLOCK**	r=0,2301<br>p=0,329	**r=0,6639**<br>**p=0,002**	**EIF2AK3 vs CLOCK**	r=0,2065<br>p=0,594	**r=0,7241**<br>**p<0,001**
**ATF4 vs CLOCK**	r=0,1950<br>p=0,410	**r=0,5877**<br>**p=0,008**	**ATF4 vs CLOCK**	r=0,3796<br>p=0,321	r=0,3125<br>p=0,325
**ATF6 vs CLOCK**	r=0,1513<br>p=0,546	r=0,2654<br>p=0,601	**ATF6 vs CLOCK**	r=0,4352<br>p=0,241	r=0,2522<br>p=0,508
**GRP78 vs CLOCK**	r=-0,1290<br>p=0,610	r=-0,1765<br>p=0,427	**GRP78 vs CLOCK**	r=-0,3768<br>p=0,317	r=-0,1691<br>p=0,663
**CHOP vs CLOCK**	r=-0,0353<br>p=0,889	r=-0,1274<br>p=0,603	**CHOP vs CLOCK**	r=-0,2683<br>p=0,485	r=-0,2763<br>p=0,471
**XBP1 vs CLOCK**	r=0,2415<br>p=0,450	r=0,359<br>p=0,323	**XBP1 vs CLOCK**	r=0,3621<br>p=0,323	r=0,0782<br>p=0,841
**NRF2 vs BMAL1**	r=0,2798<br>p=0,232	r=0,2805<br>p=0,244	**NRF2 vs BMAL1**	r=0,4447<br>p=0,230	r=0,1019<br>p=0,792
**EIF2AK3 vs BMAL1**	r=0,3068<br>p=0,2014	r=0,2439<br>p=0,314	**EIF2AK3 vs BMAL1**	r=0,2781<br>p=0,468	r=0,1434<br>p=0,712
**ATF4 vs BMAL1**	r=0,2604<br>p=0,2674	r=0,3826<br>p=0,105	**ATF4 vs BMAL1**	r=0,2577<br>p=0,503	r=0,4741<br>p=0,186
**ATF6 vs BMAL1**	r=-0,0309<br>p=0,900	r=-0,3804<br>p=0,107	**ATF6 vs BMAL1**	r=-0,0382<br>p=0,930	r=-0,2613<br>p=0,487
**GRP78 vs BMAL1**	r=-0,2332<br>p=0,351	r=-0,3562<br>p=0,299	**GRP78 vs BMAL1**	r=-0,5557<br>p=0,120	r=-0,4881<br>p=0,1825
**CHOP vs BMAL1**	r=-0,0523<br>p=0,831	r=-0,1150<br>p=0,6293	**CHOP vs BMAL1**	r=-0,1273<br>p=0,744	r=-0,0465<br>p=0,905
**XBP1 vs BMAL1**	r=-0,0146<br>p=0,952	r=-0,3543<br>p=0,136	**XBP1 vs BMAL1**	r=-0,0265<br>p=0,945	r=-0,1356<br>p=0,712
**NRF2 vs CRY1**	r=0,2795<br>p=0,232	r=0,3659<br>p=0,102	**NRF2 vs CRY1**	r=0,1812<br>p=0,572	r=0,2541<br>p=0,508
**EIF2AK3 vs CRY1**	r=0,3068<br>p=0,2014	r=0,1361<br>p=0,578	**EIF2AK3 vs CRY1**	r=0,1180<br>p=0,714	r=0,1323<br>p=0,734
**ATF4 vs CRY1**	r=0,2604<br>p=0,267	r=0,2523<br>p=0,254	**ATF4 vs CRY1**	r=0,0433<br>p=0,893	r=0,6062<br>p=0,085
**ATF6 vs CRY1**	r=0,0309<br>p=0,900	r=0,1506<br>p=0,538	**ATF6 vs CRY1**	r=0,0818<br>p=0,800	r=0,4722<br>p=0,199
**GRP78 vs CRY1**	r=-0,2805<br>p=0,244	r=0,4424<br>p=0,057	**GRP78 vs CRY1**	r=-0,2621<br>p=0,235	r=0,2865<br>p=0,419
**CHOP vs CRY1**	r=-0,2439<br>p=0,314	r=0,4377<br>p=0,060	**CHOP vs CRY1**	r=-0,2917<br>p=0,352	r=0,4140<br>p=0,265
**XBP1 vs CRY1**	r=0,402<br>p=0,204	r=0,2012<br>p=0,408	**XBP1 vs CRY1**	r=0,4758<br>p=0,195	r=0,4502<br>p=0,224
**NRF2 vs PER1**	r=0,3598<br>p=0,130	r=0,1142<br>p=0,641	**NRF2 vs PER1**	r=0,4781<br>p=0,192	r=0,3081<br>p=0,412
**EIF2AK3 vs PER1**	r=0,2239<br>p=0,356	r=0,1587<br>p=0,503	**EIF2AK3 vs PER1**	r=0,4607<br>p=0,218	r=0,4752<br>p=0,198
**ATF4 vs PER1**	r=0,3770<br>p=0,101	r=0,2411<br>p=0,320	**ATF4 vs PER1**	r=0,3400<br>p=0,247	r=0,3829<br>p=0,308
**ATF6 vs PER1**	r=0,0628<br>p=0,798	r=0,1887<br>p=0,439	**ATF6 vs PER1**	r=0,3831<br>p=0,308	r=0,0417<br>p=0,915
**GRP78 vs PER1**	r=0,4117<br>p=0,071	r=0,091<br>p=0,708	**GRP78 vs PER1**	r=0,4523<br>p=0,221	r=0,1690<br>p=0,664
**CHOP vs PER1**	r=0,0233<br>p=0,924	r=0,0869<br>p=0,723	**CHOP vs PER1**	r=0,3049<br>p=0,425	r=0,1339<br>p=0,738
**XBP1 vs PER1**	r=0,2629<br>p=0,262	r=0,1643<br>p=0,501	**XBP1 vs PER1**	r=0,2317<br>p=0,548	r=0,1123<br>p=0,773
